# Designing cross-sector leadership dialogue for successful aging: a forum-based case study and preliminary participant evaluation

**DOI:** 10.3389/fpubh.2026.1951537

**Published:** 2026-10-15

**Authors:** Myrto Pyrrou, Craig S. Anderson, Hariklia Proios

**Affiliations:** 1 Department of Educational and Social Policy, University of Macedonia, Thessaloniki, Greece; 2 Institute for Science and Technology for Brain-inspired Intelligence, Fudan University, Shanghai, China; 3 The George Institute for Global Health, University of New South Wales, Sydney, NSW, Australia; 4 Neurology Department, Royal Prince Alfred Hospital, Sydney, NSW, Australia; 5 Department of Communication Sciences and Disorders, Adelphi University, Garden City, NY, United States

**Keywords:** brain health, community case study, cross-sector dialogue, healthy aging, implementation barriers, knowledge translation, public health leadership, successful aging

## Abstract

**Background:**

Translating evidence on brain health and healthy aging into coordinated action is challenging because responsibility is distributed across health care, communities, institutions, and policy. An interdisciplinary forum was developed as a university-based pilot educational workshop to bring different professional and sectoral perspectives into structured discussion of barriers to applying knowledge in practice.

**Methods:**

We describe the design, implementation, and preliminary evaluation of the full-day Leadership Forum on Successful Aging and Longevity, held at Adelphi University on April 24, 2026. Thirty-five people attended, including three organizers, five additional invited contributors, and 27 general attendees. The program moved from personal reflection and scientific input to moderated cross-sector dialogue, facilitated diagnostic group work, structured report-back, and leadership-oriented reflection. The descriptive evaluation drew on anonymous opening reflection cards, Sense-Making Workshop flipcharts, facilitator-recorded notes from the leadership activity, and an anonymous post-forum questionnaire completed by 19 of the 27 general attendees.

**Results:**

All 19 questionnaire respondents selected the most favorable response for the venue, organization and planning, timing and duration, and willingness to attend a future forum. Eighteen selected the most favorable responses for presentations, opportunities for interaction, and overall experience. Opening reflections associated aging well with independence, health, family, support, and quality of life. Workshop outputs highlighted fragmented services, continuity and coordination of care, affordability, everyday environments, budgets, incentives, and tensions between prevention and existing economic or organizational arrangements. Open-ended feedback also identified practical areas for refinement, including room audio, microphone quality, and access to presentation materials.

**Conclusion:**

The forum was well received by respondents, with materials reflecting discussion of aging well that extended beyond health care and individual choices to relationships, services, environmental conditions, and organizational responsibility. The case provides a practical example of how scientific input, cross-sector dialogue, diagnostic group work, and leadership-oriented reflection can be combined within a one-day forum. The findings are descriptive and context-specific and do not demonstrate learning, behavior change, sustained collaboration, or longer-term impact. Future iterations could involve older adults and relevant community organizations more directly and plan follow-up from the outset where continued dialogue or action is intended.

## Introduction

Healthy aging depends not only on the availability of evidence, but also on how that evidence is understood and applied across the settings that shape later life. Prevention, brain health, social participation, and supportive environments involve responsibilities that extend beyond health care alone. Research on deliberative dialogue and cross-organizational collaboration suggests that bringing different perspectives together can be useful, although the process depends on facilitation, relationships, shared purpose, and organizational context (1–3). The Leadership Forum on Successful Aging and Longevity was developed in response to this challenge as a university-based pilot educational workshop combining scientific input, discussion across professional and sectoral perspectives, and structured group reflection.

### Contextualizing brain health within healthy and successful aging

Population aging is reshaping health systems, communities, families, and public priorities worldwide. In 2020, there were more people aged ≥60 years than children younger than 5 years (4). By 2030, one in six people will be aged ≥60 years, and by 2050 the global population in this age group is projected to reach 2.1 billion (4). These demographic changes raise a central public health question: whether gains in longevity are also accompanied by health, autonomy, dignity, participation, and meaningful social connection. This concern is consistent with Sustainable Development Goal 3, which promotes health and wellbeing at all ages (5). The challenge is therefore not simply longer life, but ensuring that later years are healthier, more equitable, and more connected.

The distinction between living longer and aging well is reflected in the concepts of healthy and successful aging. The World Health Organization (WHO) conceptualizes healthy aging around the maintenance of functional ability and wellbeing in later life (6). Early accounts of successful aging emphasized avoidance of disease and disability, maintenance of physical and cognitive function, and continued engagement with life (7). Rowe and Kahn later called for greater attention to social factors and a life-course perspective within the concept of successful aging (8). This broader view recognizes that later life is shaped not only by health status, but also by relationships, services, opportunities, and the wider environment (6).

Brain health is central to this broader understanding because it supports independence, communication, movement, emotional wellbeing, decision-making, and participation. The WHO describes brain health as multidimensional, encompassing cognitive, sensory, social-emotional, behavioral, and motor functioning across the life course (9). Brain health is therefore both a clinical concern and a life-course public health issue: protecting it begins before old age and depends on prevention, health literacy, supportive environments, and systems that help people maintain function and connection throughout life (10).

Many barriers affecting older adults are embedded in the organization of services and communities rather than arising solely from individual behavior or clinical need. Fragmented care, limited continuity between services, unequal access to prevention, social isolation, and age-related stigma can weaken support in later life (11–13). Integrated care approaches emphasize person-centered assessment, functional ability, and coordinated support across community and primary care settings (12). Research on collaboration between health and non-health organizations similarly indicates that cross-sector work depends on shared purpose, relationships, resources, governance, leadership, and local context (3).

The United Nations Decade of Healthy Aging (2021–2030) reflects this wider perspective by presenting healthy aging as a shared agenda involving governments, health and social care systems, civil society, academia, communities, the private sector, and older people themselves (14). This agenda depends on coordinated action across sectors. Against this background, the present community case study describes the context, design, implementation, and preliminary evaluation of the Leadership Forum on Successful Aging and Longevity.

## Background and rationale

The forum's design rested on a cross-sector understanding of successful aging. Later life is shaped by health systems and prevention, but also by social participation, access to services, community environments, policy decisions, business practices, and public attitudes. Bringing these perspectives together was intended to help participants consider where responsibilities intersect and where existing arrangements may support or constrain aging well (14).

Within this context, the Leadership Forum on Successful Aging and Longevity was developed as a pilot initiative to bring complementary perspectives into structured discussion around brain health and healthy aging. Its immediate purpose was to draw attention to these issues, provide a shared scientific starting point, and explore barriers to applying knowledge in practice across different settings. The forum was not designed to demonstrate changes in behavior, professional practice, or sustained collaboration, but to test a structured format for dialogue and reflection and obtain preliminary participant feedback.

A further design choice was to move beyond a lecture-only format. The program sequence was developed pragmatically by the organizing team rather than from a single formal theoretical or pedagogical framework. Its two overarching themes, “Living Longer vs. Living Well” and “From Knowledge to Impact,” reflected the intended progression of the day. The first was intended to focus attention on the distinction between longevity and aging well and to provide a shared conceptual and scientific starting point. The second shifted attention toward implementation challenges, system-level considerations, and the practical application of knowledge. Scientific input was followed by multi-perspective dialogue, facilitated group discussion, and future-oriented reflection, giving participants opportunities to examine tensions between evidence and practice and consider what coordinated action might involve. This staged approach is consistent with research emphasizing the value of facilitated dialogue when evidence and contextual knowledge are brought together (1, 15).

In this case, leadership was treated as a practical rather than positional concept. It referred to recognizing system gaps, bringing different perspectives into conversation, and reflecting on shared responsibility for more coordinated action. This was relevant to challenges such as fragmented services, unequal access to prevention, social isolation, and agism, which extend beyond clinical care and require responses across settings and sectors (11–13). Leadership capacity itself was not assessed as an outcome of the forum; this framing is not intended as a formal theoretical model of leadership.

The forum was not designed or evaluated as a formal knowledge-translation intervention. Rather, knowledge translation represented a practical concern underlying the program: the difficulty of moving from available evidence to action across settings with different responsibilities, constraints, and priorities. The term “sense-making,” used for one of the facilitated workshops, referred to the structured group process through which participants discussed and identified perceived gaps, constraints, and system-level tensions; the activity was not based on a formal sensemaking framework.

Adelphi University provided the institutional setting for the pilot following approval of the forum proposal. The university contributed the venue, organizational and logistical infrastructure, academic environment, and public channels for promotion and open registration. Research in aging-related initiatives has shown that universities can support knowledge diffusion and cross-sector collaboration, although the forum was not designed around a formal intermediary-organization model (16).

The forum was implemented as a 1-day event and is presented as a community case study of a real-world, university-based initiative, documenting its setting, design, implementation, workshop outputs, preliminary participant evaluation, and practical lessons for similar initiatives (17).

### Description of the case and essential programmatic elements

#### Forum setting, contributors, and recruitment

The Leadership Forum on Successful Aging and Longevity was a full-day, in-person event held at Adelphi University's Angello Alumni House on April 24, 2026, from 09:00 to 16:00, and hosted by the Department of Communication Sciences and Disorders in the Ruth S. Ammon College of Education and Health Sciences. Hariklia Proios organized and moderated the forum. Craig Anderson and Myrto Pyrrou served as forum co-coordinators and workshop co-facilitators, contributing to the program structure and agenda, workshop flow, participant materials, and delivery of the participatory sessions. Thirty-five people attended the forum: three organizers, five additional invited contributors, and 27 general attendees. Detailed demographic and professional characteristics were not systematically collected for the full attendee group.

Invited contributors were identified through the professional networks of the organizing team and selected purposively on the basis of professional roles, relevant expertise, and ability to contribute complementary perspectives to the forum themes. The selection sought to include expertise in stroke and brain health, cardiovascular prevention, public health implementation, advocacy and patient support, business and innovation, and organizational leadership. No formal systematic search or scoring procedure was used.

The core organizing team held several preparatory meetings to develop and refine the agenda, workshop sequence, guiding questions, participant materials, and contributor roles. Invited contributors were coordinated by email, joined a collective online briefing 2 weeks before the forum to review the program and their roles, and received a final on-site briefing. Registration was open to interested attendees rather than targeted stakeholder groups and promoted through the College and Department channels, including the event page and on-campus flyers; no formal eligibility criteria applied.

### Program structure and evidence-to-dialogue sessions

The program moved from initial reflection and scientific input to expert dialogue, facilitated group work, structured reporting, and leadership-oriented reflection. The two overarching themes, “Living Longer vs. Living Well” and “From Knowledge to Impact,” provided the organizing structure for this progression. The sequence, duration, and intended function of activities are summarized in Table 1.

**Table 1:** Structure of the leadership forum on successful aging and longevity.

Time	Activity	Lead/facilitator (s)	Intended function
09:00–09:10	Administrative welcome and purpose of the forum	University and forum leadership	Introduce the forum and its purpose
09:10–09:15	Audience reflection: “what does aging well mean?”	Hariklia Proios	Surface initial participant associations with aging well
09:15–09:45	Keynote: “perspectives on maintaining longevity and brain health: the facts”	Craig Anderson	Provide scientific input on longevity and brain health
09:45–10:10	Moderated Q&A	Craig Anderson; Hariklia Proios	Extend discussion of longevity, quality of life, and brain health
10:10–10:40	Coffee break	–	–
10:40–10:45	Introduction of speakers	Chris Macey	Introduce the expert dialogue
10:45–11:15	Expert dialogue	Invited contributors; moderated by Chris Macey	Consider implementation and cross–sector responsibility
11:15–12:20	Sense–making workshop	Hariklia Proios; Myrto Pyrrou; invited facilitators	Identify gaps, structural constraints, and system–level tensions
12:20–13:20	Lunch	–	–
13:20–13:50	Structured report–Back	Hariklia Proios; Myrto Pyrrou	Share thematic group outputs with the full forum
13:50–13:55	Reflections from speakers	Invited contributors and facilitators	Briefly reflect on the group discussions
13:55–15:00	Leadership workshop: “creating the biggest possibility”	Hariklia Proios; Craig Anderson	Support future–oriented leadership reflection
15:00–15:30	Participant sharing	Hariklia Proios; Craig Anderson	Share individual reflections from the leadership activity
15:30–16:00	Overview and final thoughts	Hariklia Proios; Craig Anderson	Close the forum and summarize the day

Following an administrative welcome, Hariklia Proios led a 5-min audience reflection in which participants wrote one word in response to the prompt, “What does aging well mean to you?”, briefly shared their responses, and displayed them in the room. The activity was intended to surface participants' initial associations with aging well before the scientific sessions.

Craig Anderson, Director of the Global Brain Health Program at The George Institute for Global Health and President-elect of the World Stroke Organization, delivered the 30-min keynote, “Perspectives on Maintaining Longevity and Brain Health: the Facts,” addressing longevity, brain health, and the importance of protecting brain health across the life course. A 25-min moderated discussion with Hariklia Proios followed, focusing on the distinction between increased longevity and living well, factors shaping quality of life in older age, and the communication of brain health earlier in life.

A 30-min expert dialogue moderated by Chris Macey, Director of Advocacy and Patient Support at the Irish Heart Foundation, brought together Socrates Kakoulides, who contributed a clinical and cardiovascular prevention perspective; Nicola Anderson, a business and innovation perspective; Jan van der Merwe, stroke prevention and public health implementation experience; and Alan Cooper, an organizational leadership and systems perspective. Guiding questions addressed barriers to implementing prevention and brain health knowledge at scale, cross-sector responsibility, and leadership needed to move from knowledge toward action. The session was intended to broaden the discussion from scientific evidence to implementation and cross-sector responsibility before the participatory workshop.

### Participatory workshops and reporting

In the 65-min Sense-Making Workshop, co-led by Hariklia Proios and Myrto Pyrrou, participants worked in three thematic groups: Health Systems and Clinical Structures; Implementation and Prevention; and Business, Policy and Cross-Sector Leadership. General attendees were distributed across the three thematic groups to maintain broadly similar group sizes; exact group counts were not retained. Contributors were allocated according to relevant expertise: Socrates Kakoulides and Craig Anderson facilitated the first group, Jan van der Merwe and Chris Macey the second, and Nicola Anderson and Alan Cooper the third. All groups used the same instruction sheet and core prompts.

The workshop was framed primarily as a diagnostic exercise. Each group was asked to discuss and agree on one key gap, one structural constraint, and one system-level tension affecting successful aging. Facilitators guided the discussion and recorded key points and the wording agreed by the group on flipcharts. Agreement was reached informally through facilitated discussion; no voting or formal consensus procedure was used. The aim was to identify practical and structural concerns rather than require formal action plans, although practical suggestions were recorded when they arose spontaneously.

After lunch, each group used its flipchart to present its gap, structural constraint, and system-level tension during a 30-min report-back to the full forum, followed by brief reflections from invited speakers. The leadership activity followed as a separate reflective exercise and did not formally require participants to build on the preceding group outputs.

The 65-min leadership workshop, “Creating the Biggest Possibility,” was led by Hariklia Proios and Craig Anderson, with additional facilitation and recording support from Myrto Pyrrou. Participants worked in pairs while completing individual guided worksheets on a desired future outcome, how success might be assessed, the leadership and team needed, and possible milestones. The worksheets were intended as personal reflection tools, retained by participants, and not collected for analysis. Facilitators instead recorded key ideas shared during discussion on flipcharts; these notes were retained for descriptive review.

A 30-min participant sharing period followed, during which participants briefly presented key points from their individual reflections, before a 30-min overview and closing. The program provided opportunities for participation through individual reflection, facilitated group discussion, report-back, and consideration of future directions and responsibilities.

## Methodological aspects

We used a community case study approach to describe the design, implementation, and preliminary evaluation of the Leadership Forum on Successful Aging and Longevity. The aim was to document a real-world, university-based initiative and consider how its structure, participatory process, and early feedback might inform similar public health and leadership-oriented initiatives on successful aging, rather than to test effectiveness or assess changes in learning, behavior, or professional practice. The evaluation was preliminary because it relied on immediate post-forum feedback and descriptive workshop materials, with no pre-forum measures, longitudinal follow-up, or additional data collection.

Thirty-five people attended the forum overall: three organizers, five additional invited contributors, and 27 general attendees. Nineteen of the 27 general attendees voluntarily completed the anonymous post-forum questionnaire and constituted the questionnaire sample, corresponding to a response proportion of 70.4%. Organizers and invited contributors were not included in the questionnaire sample. Planning documents and the participant program were used to document the forum's structure and implementation. The preliminary evaluation drew on four sources: anonymous opening reflection cards, Sense-Making Workshop group flipcharts, facilitator-recorded flipchart notes from the leadership activity, and anonymous post-forum questionnaires. Individual leadership worksheets were retained by participants and were not collected or analyzed. The Sense-Making flipcharts were group-level outputs, while the leadership notes captured ideas shared during discussion without identifying contributors. Reflection cards informed the findings on initial meanings of aging well; Sense-Making flipcharts informed the findings for the three thematic domains; leadership notes informed the leadership-oriented reflections; and questionnaires provided the post-forum evaluation findings. These materials were used descriptively and not as evidence of how widely particular views were held.

At the end of the forum, general attendees were invited to complete an anonymous paper-based questionnaire when collecting their certificates of participation. Participation in the evaluation was voluntary, and receipt of a certificate did not depend on questionnaire completion. Those who chose to participate completed the questionnaire independently in a separate area and returned it before leaving the venue. All 19 returned questionnaires contained an entry for every item.

The questionnaire contained nine closed-ended items and two open-ended items; the full item wording, response options, and response distributions are presented in Table 2. The respondent-category item recorded participants' relationship to the university; one respondent self-identified as staff. No additional demographic or professional information, including age, gender, professional field, sector, or leadership experience, was collected. Closed-ended responses were summarized using counts; no inferential analyses were conducted because of the small, voluntary, self-selected sample.

**Table 2:** Post-forum questionnaire items and response distribution (*n* = 19).

Questionnaire item	Response distribution, *n*	Missing, *n*
1. Are you:	Current student: 2; faculty: 6; alumni: 4; visitor: 6; staff^*^: 1	0
2. Did you find the venue suitable for the event?	Yes, it was perfect: 19; It was okay: 0; No, it was not suitable: 0	0
3. How would you rate the organization and planning of the event?	Very well organized: 19; Somewhat organized: 0; needs improvement: 0	0
4. Was the time, day of the week, and duration of the event convenient for you?	Yes, the time, day of the week, and duration were convenient: 19; time and day of the week, were convenient, but duration was too long/short: 0; Time and day of the week were inconvenient: 0	0
5. Did you find the presentations informative and engaging?	Yes, very engaging: 18; somewhat engaging: 1; not engaging at all: 0	0
6. How would you rate the networking opportunities provided at the forum?	Excellent: 10; good: 9; fair: 0; poor: 0	0
7. Were there enough opportunities to interact with other attendees and organizers?	Yes, plenty: 18; It was okay: 1; no, there could have been more opportunities: 0	0
8. In the future would you attend future forums organized by the Ruth Ammon College of Education and Health Sciences?	Definitely: 19; maybe: 0; No: 0	0
9. How would you rate your overall experience at the forum?	Excellent: 18; good: 1; average: 0; poor: 0	0
10. “Were there any specific activities or aspects of the event that you particularly enjoyed?”^**^	Comment provided: 16; N/A: 3	0
11. “What aspects of the forum do you think could be improved for future events?”^**^	Specific suggestion or concern: 7; no specific improvement indicated or satisfactory as delivered: 12	0

^*^“Staff” was not a predefined response option; one respondent self-identified as staff. ^**^For the open-ended items, “N/A” and responses indicating no suggested improvement were treated as responses rather than missing data.

The first author conducted the initial descriptive review of the open-ended questionnaire responses, opening reflection cards, Sense-Making Workshop flipcharts, and facilitator-recorded leadership notes. All retained materials were read in full, and recurring ideas and practical concerns were noted. The other authors reviewed the material and agreed on the issues reported. No formal coding framework, thematic analysis, or qualitative analysis software was used. Because the workshop records were not individual-level transcripts, frequency estimates and subgroup comparisons were not attempted. The materials were used descriptively to identify issues related to aging well, system gaps, implementation challenges, and leadership responsibilities, rather than to support claims about participant learning, consensus, representativeness, behavioral change, or longer-term outcomes.

The broader program within which the forum was conducted was reviewed by the Adelphi University Institutional Review Board and received an exempt determination (IRB No. 20250097). Participation in the feedback process was voluntary, and the questionnaire was completed anonymously. Participants were informed that their anonymous responses could be used for research and reporting purposes. No identifying personal information was collected through the questionnaire, reflection cards, workshop flipcharts, or facilitator-recorded leadership notes.

## Results

### Respondent profile and post-forum evaluation

Nineteen of the 27 general attendees (70.4%) completed the post-forum questionnaire and were included in the preliminary evaluation. Respondents included six faculty members, six visitors, four alumni, two current students, and one respondent who self-identified as a staff member. There were no missing responses to the closed-ended questionnaire items. The full distribution of responses is presented in Table 2.

Responses were concentrated in the most favorable categories. All 19 respondents considered the venue suitable, rated the event as very well organized, and reported that the time, day of the week, and duration were convenient. All 19 also indicated that they would definitely attend a future forum. Eighteen respondents described the presentations as very engaging, with one selecting somewhat engaging. Similarly, 18 reported that there were plenty of opportunities for interaction, while one considered the opportunities adequate. Overall experience was rated as excellent by 18 respondents and good by one. Networking opportunities were rated as excellent by 10 respondents and good by nine.

Sixteen respondents provided a written response to the open-ended question asking which activities or aspects of the event they particularly enjoyed; three entered “N/A.” Comments referred to the keynote and other speakers, the expertise and perspectives represented on the panel, the breakout-group format, and opportunities to hear and discuss ideas with other participants. One respondent described the breakout groups as “a nice way to interact and explore topics,” while another highlighted the “knowledge and expertise of the panel and organizing team.” Other comments referred to the forum as thought-provoking and to the value of hearing research and perspectives from international contributors.

Seven respondents provided a specific suggestion or concern in response to the question about what could be improved in future events. These included problems with room audio or microphone quality, access to presentation slides in a larger format or electronically, and interest in additional content related to aging and cognitive decline, hearing, and speech-language pathology. One respondent noted that, although time for interaction was available, attendees did not interact much away from their tables, while another suggested making the forum longer. The remaining responses either indicated no specific improvement or stated that the event had been satisfactory as delivered.

### Initial meanings of aging well

During the opening audience reflection, participants recorded one word or a short phrase describing what aging well meant to them. Responses recorded on the reflection cards included independence, health, family, support, happiness, energy, quality of life, fulfillment, productivity, and strength. Among the responses were individually oriented ideas, such as health, independence, and strength, as well as relational ideas, including family and support.

### Sense-making workshop outputs

The three Sense-Making groups worked from the same prompts, although the flipcharts varied in how explicitly they separated gaps, structural constraints, tensions, and possible responses. The Health Systems and Clinical Structures group focused on care pathways, workforce, continuity of care, and integration of services. Its flipchart described a disjointed health care system and recorded an electronic or common medical system and patient-owned medical records as possible responses to fragmentation. Economic constraints were also noted, together with a greater emphasis on prevention and wellness and on relationship-centered care.

The Implementation and Prevention group focused more directly on everyday conditions affecting preventive behavior. Its flipchart referred to access to unhealthy food, the relative affordability of unhealthy options, sedentary lifestyles, and the role of technology and convenience services in facilitating access to less healthy choices. Health-related stigma was recorded as a structural constraint, alongside the perceived need for individuals to advocate for themselves. Although the workshop did not require formal solution development, several practical suggestions arose during this discussion, including making healthier foods more affordable, using higher taxation to discourage unhealthy options, reducing the availability of unhealthy foods in the home, and identifying existing “bright spots” in implementation.

The Business, Policy and Cross-Sector Leadership group emphasized economic and system incentives. Its flipchart referred to budgets and return on investment, socioeconomic differences in knowledge across communities, environmental influences, and the role of incentives in shaping prevention. The group also recorded a tension between prevention and financial interests, with notes referring to potential financial losses in areas such as the soda industry, medications, and clinical services. A prevention-oriented culture and incentives that support preventive action were also discussed.

The three group outputs therefore emphasized different aspects of the implementation challenge. The health systems discussion centered mainly on continuity and coordination of care; the implementation and prevention discussion focused on everyday environments and behavioral conditions; and the business and policy discussion placed greater emphasis on incentives, resource allocation, and tensions between prevention and existing economic or organizational arrangements. These outputs represent the issues documented by the three groups and should not be interpreted as indicating how widely each view was shared among forum attendees.

### Leadership-oriented reflections

The facilitator-recorded notes from the leadership activity captured ideas that participants shared during the discussion rather than the contents of their individual worksheets, which were not collected. The recorded ideas included stronger community ties and intergenerational engagement, more accessible health and wellness services, mental health support, preventive care, and greater recognition of older adults' knowledge and contributions to community life. The notes also referred to ways of moving from broad aspirations toward action, including identifying measures of progress, forming teams or coalitions, and considering incentives that could support prevention.

These notes therefore provide examples of ideas shared during the leadership discussion, rather than a complete record of each participant's reflection.

## Discussion

Across the forum materials, aging well was discussed as more than a clinical or individual issue. Brain health provided the initial scientific focus, but the reflection cards and workshop outputs extended the discussion to independence, family and social support, access to services, prevention, affordability, environmental influences, and institutional and leadership responsibilities. This broader perspective is consistent with the framework presented by Beard et al. (18), in which healthy aging is understood through functional ability and the interaction between individual capacity and the environments in which people live. It also reflects critiques of traditional successful aging models, particularly concerns about their emphasis on disease avoidance, individual achievement, and personal responsibility at the expense of social, economic, and structural conditions (19). The materials therefore reflected discussion across personal meanings, everyday environments, service organization, and wider system-level conditions, without indicating consensus among participants.

The forum's staged structure moved from scientific input and moderated expert dialogue to diagnostic group discussion, structured report-back, and leadership-oriented reflection. Respondents generally evaluated the forum positively, and the participatory activities generated documented contributions. On that basis, the forum met its immediate purpose of piloting a structured format for discussion, exploring barriers to applying knowledge in practice, and obtaining preliminary feedback. The approach shares features with facilitated stakeholder and deliberative dialogue, where evidence is considered alongside experiential and contextual knowledge (1, 15). Focused prompts and facilitation were intended to keep the participatory sessions centered on shared questions while providing opportunities for different perspectives to be expressed. The format is therefore best viewed as a way of structuring dialogue rather than as evidence of intervention effectiveness.

The Sense-Making Workshop highlighted implementation challenges across care coordination, everyday environments and preventive behavior, and organizational or economic arrangements. Although the group outputs cannot indicate how widely particular views were shared, they are consistent with implementation research showing that moving evidence into practice involves more than dissemination alone. Rycroft-Malone et al. (20) found that collective implementation depends on relationships and the alignment of vision, values, structures, and processes across organizations. Greenhalgh et al. (2) similarly emphasize the importance of context, relationships, governance, and facilitation when knowledge is developed or applied with stakeholders. The forum did not resolve these barriers, but the outputs illustrate how implementation problems can be considered at service, behavioral, environmental, and organizational levels.

Leadership was treated in practical terms. The leadership activity and facilitator-recorded notes referred to forming teams or coalitions, identifying measures of progress, strengthening community connections, and considering incentives that could support prevention. Invited contributors brought clinical, academic, public health, advocacy, business, and organizational perspectives into the discussion, reflecting how responsibility for healthy aging can extend across services, communities, institutions, and policy. These reflections do not demonstrate the development of leadership capacity; rather, they show how leadership was used to consider shared responsibility when challenges extend beyond a single profession or organization. Adelphi University provided the setting and infrastructure for bringing these perspectives together. Research in age-friendly initiatives has described broader intermediary functions for universities, including facilitating cross-sector collaboration and knowledge diffusion (16). Here, the university's role was narrower and primarily convening. Evidence on collaboration between health and non-health organizations indicates that sustained collaboration depends on shared purpose, relationships, resources, governance, leadership, and external context, while evidence of downstream health effects remains limited (3). A 1-day forum is therefore better understood as an opportunity for cross-sector dialogue than as an established collaborative structure.

The participatory elements overlap in some respects with community-engaged approaches through facilitated discussion, participant-generated concerns, and perspectives from different professional and organizational settings. However, the forum was not designed as community-based participatory research (CBPR) or as a co-design process. Older adults were not recruited as a distinct partner group, and participants did not share governance of the research process or participate in an ongoing community partnership. CBPR, by contrast, involves sustained collaboration between researchers and community members throughout the research process (21). The initiative is therefore better described as a participatory forum with some community-engaged features.

The post-forum questionnaire provides evidence primarily about acceptability among those who chose to complete it. Ratings were concentrated in the most favorable categories, while open-ended responses identified areas for refinement. These findings should not be interpreted as evidence of learning or longer-term impact. The contribution of this case is mainly practical and descriptive rather than methodological novelty: it documents how established dialogue and workshop components were combined in a healthy-aging forum to move from scientific input to diagnostic discussion across service, environmental, and organizational levels. The process may be relevant to similar settings, although whether it produces comparable engagement or useful outputs elsewhere remains to be examined.

### Lessons learned and recommendations

One practical lesson concerns the sequencing of activities. The forum moved from scientific input and moderated dialogue to diagnostic group work, structured report-back, and only then to leadership-oriented reflection. For similar initiatives, separating the identification of gaps, structural constraints, and system-level tensions from later consideration of possible responses may help preserve a diagnostic focus. In practice, however, the flipcharts did not always distinguish these categories clearly, and practical suggestions sometimes arose during diagnostic discussion. Common prompts, clear instructions across groups, facilitators with relevant expertise, and flipcharts used to record agreed points provided a clear structure for diagnostic discussion and report-back. These features may be useful when participants bring different professional and personal perspectives to the same issue. This emphasis on structure and facilitation is consistent with stakeholder and deliberative dialogue literature, which describes dialogue as a structured process and highlights the role of moderation (1, 15).

Participant feedback identified practical areas for refinement, including room audio and microphone quality, the visibility and availability of presentation materials, additional topics, and interaction beyond the thematic tables. One respondent suggested a longer forum, while another noted limited interaction away from the tables despite time being available; closed-ended ratings of duration and opportunities for interaction were nevertheless positive. Rather than adding unstructured networking time, future forums could build in more deliberate opportunities for interaction across tables or groups. Audiovisual equipment and slide visibility should be checked in advance, and electronic access to presentation materials may improve accessibility. Beyond these participant suggestions, future iterations should also involve older adults and organizations representing them more directly, ideally from the planning stage, so that their perspectives and lived experiences can inform the agenda and discussion.

The present forum had no predefined follow-up or implementation component. Where continued dialogue or action is intended, follow-up should therefore be planned from the outset rather than added after the event. Options could include circulating a short summary, convening a targeted webinar or smaller follow-up meeting, and assigning responsibility for realistic next steps, with any plan kept proportionate to available time and resources. Previous work on deliberative dialogue has highlighted the importance of post-event communication, leadership, and resources for follow-up, while collaborative implementation research emphasizes the importance of relationships and processes that develop over time (1, 20).

### Conceptual and methodological constraints

Successful aging is not a neutral or universally agreed term and can overlook inequalities, illness, disability, and diverse experiences in later life (19). In this forum, the term served as a broad frame for discussion rather than as an evaluative standard for older adults. The participatory elements should not be taken as evidence of co-creation, consensus, or an established cross-sector partnership. Older adults were not recruited as a distinct stakeholder group, so the forum outputs cannot be taken as a direct representation of their priorities or lived experiences.

This was a single-site, 1-day case study in a university setting. The questionnaire was based on 19 voluntary post-forum responses and may not reflect all attendees' views. The questionnaire was developed specifically for this event and was not a validated evaluation instrument. The voluntary sample may have been subject to self-selection and social-desirability bias, with responses concentrated in the highest categories, limiting differentiation at the upper end of the scales. Beyond respondents' relationship to the university, no demographic or professional information, such as age, gender, professional field, sector, or leadership experience, was collected, limiting the characterization of the evaluation sample and preventing subgroup comparisons. Without pre-forum measures or longitudinal follow-up, the evaluation cannot support conclusions about changes in learning, attitudes, behavior, collaboration, or longer-term outcomes. The group-level materials did not capture individual contributions, minority disagreement, or how widely particular views were shared. The authors' involvement in the forum's design, facilitation, and interpretation may have shaped the account presented. The findings should therefore be understood as descriptive and context-specific, although the account may still inform similar initiatives in other settings.

## Conclusions

This case illustrates how a university-based, deliberately staged forum can combine scientific input, cross-sector dialogue, diagnostic group work, and leadership-oriented reflection in a shared discussion on healthy aging. Preliminary feedback indicated that the forum was well received by respondents, while the forum materials reflected discussion of aging well that extended beyond health care and individual choices to relationships, environments, services, policy, and institutional responsibility. Future iterations could involve older adults and relevant community organizations more directly and plan follow-up from the outset, particularly where continued dialogue or action is an intended goal.

## Data Availability

The raw data supporting the conclusions of this article will be made available by the authors, without undue reservation.
